# Editorial: The neurobiology of values

**DOI:** 10.3389/fneur.2024.1377129

**Published:** 2024-03-06

**Authors:** Niall Kavanagh, Caroline Prioleau, Bruce Miller

**Affiliations:** University of California, San Francisco (UCSF) Memory and Aging Center, Global Brain Health Institute, San Francisco, CA, United States

**Keywords:** values, neurobiology, brain health, prosocial values, neurological disorders, cross-cultural perspectives, social behavior

In recent years, the study of neurology and its implications for health equity has undergone significant development and progress. As our understanding of the brain continues to evolve, there's a growing desire to better understand the biology of social behavior and to integrate prosocial values into neurology research and practice. As editors, our aim is to explore fundamental questions: What do we mean by “values,” and how do they impact the brain? The resulting collection of articles, titled “*The neurobiology of values*,” takes a profound dive into how our brains shape and influence our values. This editorial aims to outline the main goals of the research in the Research Topic, summarizing important discoveries and putting them into a broader perspective.

## Social engagement and brain health

In their perspective, “One step beyond the lab and clinic: “walking the dementia conversation””, Zegarra-Valdivia et al. take a bold step beyond the lab and clinic bringing together researchers, caregivers, and patients to “walk the talk” for dementia. This article not only shifts the focus from pathology to conversation but also emphasizes the importance of community engagement and real-world discussions in shaping our understanding of brain health and values.

Fitri et al. highlight the profound impact of empathy as a crucial skill in disrupting disparities in global brain health. This research advocates for a collective approach to address global mental health challenges, transcending geographical and cultural boundaries.

Ibanez et al. pose a compelling question in “Can prosocial values improve brain health*?*” Here, the focus shifts to the symbiotic relationship between prosocial values and the wellbeing of the brain, holding promises for advancing preventative and intervention strategies.

Berendzen's exploration of social attachment as a window into the neural basis of prosocial values offers valuable insights into the emotional underpinnings of our social behaviors. This research contributes to the growing body of knowledge surrounding empathy, cooperation, and the neural substrates that facilitate prosocial behavior.

## Cultural perspectives on brain health

Stirland et al. invite us to consider the cultural basis of authenticity and leadership in brain health, prompting reflections on how cultural nuances influence cognitive wellbeing. The article further examines how societal values, when aligned with principles of respect and equity, contribute to overall cognitive health.

Hill-Jarrett invites us to explore “The Black radical imagination: a space of hope and possible futures,” encouraging a departure from conventional perspectives. The cultural and social dimensions explored in this article inspire a reevaluation of the intersections between culture, identity, and brain health, challenging us to recognize the rich diversity of experiences that shape our values.

## Person-centered approaches in brain health

Merrilees et al. shift attitudes toward aging, dementia, and caregiving by using personal narrative to promote person-centered values. Through the integration of personal stories, this research transcends traditional academic boundaries, providing a humanistic perspective on brain health and values.

Kaczmarska adds a unique rhythm to the Research Topic. Through the lens of dance, this research perspective not only offers insights into potential therapeutic avenues for those with dementia but also underscores the profound connection between embodiment and the brain. It highlights the significance of physical experiences in shaping our cognitive landscapes and the potential therapeutic benefits of embodied practices in promoting brain health and wellbeing.

## Values and neurological disorders

Antoniou et al. explore the intricate moral landscapes of individuals grappling with frontotemporal dementia (FTD). This research delves into the ethical dimensions of cognitive challenges, urging us to reconsider societal structures and support systems for those navigating the complex terrain of moral decision-making amid neurodegenerative conditions.

A unique dimension is added to the Research Topic through the investigation of neuroanatomical and cellular degeneration in patients with social disorders characterized by new ritualistic belief systems (Ohm et al.). This comparative study between a TDP-C patient and a Pick patient sheds light on the intricate relationship between neural degeneration and social disorders. Understanding these connections may pave the way for innovative interventions and treatments in the realm of mental health.

## Personality traits and neurobiology

In their exploration, Abu Raya, Ogunyemi, Broder et al. shed light on individual differences in personality traits with underlying neurobiological foundations. This piece expands our comprehension of how traits such as openness contribute to the diversity of values and cognitive processes, shaping our unique interactions with the world.

The reciprocal relationship between openness and creativity is a subject explored by Abu Raya, Ogunyemi, Rojas Carstensen et al.. From neurobiological perspectives to the influence of multicultural environments, the authors traverse the complex interplay between personality traits and neural processes. This research not only broadens our understanding of individual differences but also highlights the interconnectedness of neural processes with psychological traits, offering insights that extend beyond the laboratory into real-world scenarios.

## Cross-cultural perspectives on respect, equity, and leadership in brain health

Khalaila et al. explore the neural underpinnings of respect across different cultures. Using a neuroscientific lens, the authors provide valuable insights into the mechanisms through which respect is processed in the brain, offering a nuanced understanding of this complex socio-cultural phenomenon.

Arshad et al. explore the impact of respect, equity, and leadership in brain health. This research extends beyond individual cognitive processes, examining the societal and organizational factors that contribute to overall brain health.

## Economic and social values in decision-making

Messimeris et al., present an intriguing exploration of economic and social values by investigating the impact of lesions to the human ventromedial prefrontal cortex. By examining the neurological consequences of such lesions, the authors shed light on the neural substrates that govern our decision-making processes in social and economic contexts. This research not only informs our understanding of the brain's role in value-based choices but also holds implications for fields like economics, where the exploration of decision-making mechanisms is key.

## Fairness and equity in brain health

The article by Avelino-Silva et al. delves into the intricate relationship between fairness and the brain. From the depths of our guts to the complexities of neural processes, the authors critically examine the neurological mechanisms that establish our sense of fairness. Their insights contribute to a deeper understanding of the cognitive processes involved in shaping our ethical values, with potential implications for fields ranging from psychology to ethics.

## Hope and positive impact on society

Dasgupta et al. inject a note of optimism with “Hope for brain health: impacting the life course and society.” By examining interventions across the life course, the research suggests that positive impacts on individuals can resonate on a societal level. This article challenges us to consider brain health as a collective responsibility, urging society to invest in strategies that promote cognitive wellbeing throughout the lifespan.

The articles in “*The neurobiology of values*” Research Topic create a fascinating picture of how our brains and values are closely connected. Each article, like a unique thread, adds something special to our understanding, whether it's about the brain's role in fairness or how our brains shape our social behavior. This Research Topic helps us see that our values aren't just abstract ideas; they're deeply woven into our brains, impacting how we think, decide, and act. It's an exciting journey through the neural pathways of human values, offering a complete view that goes beyond labs and clinics into the diverse landscapes of culture, society, and our everyday experiences.

## Author contributions

NK: Writing—original draft, Writing—review & editing. CP: Writing—original draft, Writing—review & editing. BM: Writing—original draft, Writing—review & editing.

